# Levosimendan limits reperfusion injury in a rat middle cerebral artery occlusion (MCAO) model

**DOI:** 10.1186/1471-2377-13-106

**Published:** 2013-08-12

**Authors:** Marc Hein, Norbert Zoremba, Chistian Bleilevens, Christian Bruells, Rolf Rossaint, Anna B Roehl

**Affiliations:** 1 Department of Anesthesiology, RWTH Aachen University Hospital, Pauwelstrasse 30, Aachen 52074, Germany

**Keywords:** Experimental stroke, Postconditioning, Levosimendan, Cerebral reperfusion injury

## Abstract

**Background:**

Neuroprotective strategies in ischemic stroke are an important challenge in clinical and experimental research as an adjunct to reperfusion therapy that may reduce neurologic injury and improve outcome. The neuroprotective properties of levosimendan in traumatic brain injury in vitro, transient global brain ischemia and focal spinal cord ischemia suggest the potential for similar effects in transient brain ischemia.

**Methods:**

Transient brain ischemia was induced for 60 min by intraluminal occlusion of the middle cerebral artery in 40 male Wistar rats under general anesthesia with s-ketamine and xylazine and with continuous monitoring of their blood pressure and cerebral perfusion. Five minutes before inducing reperfusion, a levosimendan bolus (24 μg kg ^-1^) was administered over a 20 minute period. Infarct size, brain swelling, neurological function and the expression of inflammatory markers were quantified 24 hours after reperfusion.

**Results:**

Although levosimendan limited the infarct size and brain swelling by 40% and 53%, respectively, no effect on neurological outcome or mortality could be demonstrated. Upregulation of *tumor necrosis factor α* and *intercellular adhesion molecule 1* was significantly impeded. Cerebral blood flow during reperfusion was significantly reduced as a consequence of sustained autoregulation.

**Conclusions:**

Levosimendan demonstrated significant neuroprotective properties in a rat model of transient brain ischemia by reducing reperfusion injury.

## Background

Reperfusion induced by systemic thrombolysis has become the standard therapy for reducing neurological injury after acute ischemic stroke. Severe side effects (e.g., cerebral bleeding), poor reperfusion rates, a limited therapeutic window and a delayed admission of patients to specialized units have resulted in an increased use of endovascular approaches, which include intraarterial lysis, thrombectomy and thromboaspiration [1]. Adjunct therapies are able to increase ischemic tolerance or limit reperfusion injury and thus may extend the therapeutic window or improve the efficiency of reperfusion therapy. Neuroprotective actions target the brain parenchyma itself or are mediated by vascular effects. For example, augmentation of collateral flow may be a promising option [2]. Hypothermia and several neuroprotective agents (e.g., glutamate antagonists, free-radical scavengers, calcium antagonists, potassium channel activators, GABA agonists, and opiate antagonists) have been investigated in preclinical studies, with none demonstrating efficiency in clinical practice [3,4]. The inodilator levosimendan should, in theory, provide neuroprotection, as it has been shown to alter important processes in the biochemical cascade, such as the expression of inducible nitric oxide synthase and mitochondrial ATP-dependent potassium (mK_ATP_) channels which lead to apoptosis or necrosis following cerebral ischemia and reperfusion [5,6]. In fact, levosimendan has been shown to reduce cell death and inflammatory responses and improve function after transient ischemia of the spinal cord in rabbits [7,8]. While a dose-dependent protective effect in an in vitro model of traumatic brain injury could be demonstrated [9], levosimendan failed to improve brain metabolism or reduce glutamate release, inflammation and dysfunction of autoregulation in the initial phase after experimental global ischemic/hypoxic cerebral injury [10]. Its effect on focal transient ischemia has not been investigated in vivo in the brain as it has been in the heart [11]. Therefore, we investigated the application of levosimendan during ischemia and early reperfusion (postconditioning) in a rat model of middle cerebral artery occlusion (MCAO) by evaluating infarct size, inflammation, cerebral perfusion and neurological function.

## Methods

### Instrumentation

All experiments followed the Guide for the Care and Use of Laboratory Animals [12] and were authorized by the German governmental animal care and use office (Landesamt für Natur-, Umwelt- und Verbraucherschutz Nordrhein-Westfalen, Recklinghausen, Germany, Protocol No. 8.87-50.10.37.09.258). Forty male Wistar rats (Charles River, Sulzfeld, Germany) weighing between 350 and 450 g (400 ± 27 g) [13] underwent MCAO according to a previously established model. For at least one week prior to surgery, the animals were housed in standard cages in a pathogen-free environment, with free access to food and water and with a 12 hour light/dark cycle. After induction of anesthesia with an intraperitoneal injection of 100 mg/kg s-ketamine (Ketanest S, Pfizer, New York, USA) and 10 mg/kg xylazine (Xylazine 2%, Medistar, Ascheberg, Germany), three ECG electrodes were placed to monitor heart rate (HR). Animals were orally intubated and mechanically ventilated in a pressure-controlled mode to maintain an end-tidal CO_2_-tension between 35 and 40 mmHg. Anesthesia was maintained by repetitive injections of 20 mg/kg s-ketamine if a positive reaction to surgical stress or intermittent tail pinch could be observed. Body temperature (BT) was adjusted to 37°C using an esophageal probe and a back-coupled heating plate (MLT1403 and TCAT-2 Controller, ML 295/R, Physitemp Instruments, New Jersey, USA). In the prone position, the left parietal bone was thinned, and a laser Doppler flow probe (VP10M200ST/P10d, Moor Instruments, Devon, UK) was fixed 5.5 mm lateral and 1 mm occipital of the bregma to measure cerebral blood flow (CBF). In the supine position, the left external jugular was cannulated to administer 3 mL kg^-1^ h^-1^ Ringer’s solution. The left common and external carotid arteries were ligated and a pressure catheter (Micro Tip SPR G71/NR, Millar instruments, Texas, USA) was threaded into the descending aorta to measure the mean arterial pressure (MAP). The same incision was used to insert a filament with a smooth surface, blunted tip and constant diameter of 300 μm (No 270, VYGON, Aachen, Germany) into the internal carotid artery (ICA).

### Experimental protocol

After stabilization and recording of baseline values for 20 min, the filament was pushed forward until a rapid drop of CBF to 20-30% of baseline values occurred. Animals were randomized into the levosimendan or the control group using a closed envelope system. Fifty-five min after induction of MCAO, the levosimendan group were treated with a bolus of 24 μg kg^-1^ levosimendan (Simdax, Orion Pharma, Espoo, Finland) and the control group was given an equal amount of saline over a 20-min period. Sixty min after MCAO, the filament was removed and parameters were recorded for an additional 30 min. After removal of the catheters, wound closure, administration of local anesthesia with 0.2% ropivacaine (Naropin, AstraZeneca, Plankstadt, Germany) and an intraperitoneal injection of 20 mg/kg metamizole (Novalgin, Sanofi Aventis, Frankfurt, Germany), the animals were extubated at the return of sufficient spontaneous breathing and the righting reflex. Twenty-four hours later, the animals were neurologically evaluated and euthanized with an intraperitoneal injection of 100 mg kg^-1^ thiopental (Trapanal, Nycomed, Konstanz, Germany). Serum samples were collected, and the brain was removed after transcardial perfusion with 100 mL ice-cold Ringer’s solution.

### Hemodynamics

All signals were continuously recorded with a data acquisition system (PowerLab®, ADInstruments, Spechbach, Germany). HR, MAP, CBF, BT and end-tidal CO_2_ were analyzed every 10 minutes. CBF values were normalized to baseline. The autoregulatory index (ARI) was calculated as the slope obtained by linear regression from the relative change in CBF and MAP approximately 20 minutes before and after MCAO.

### Neurological injury

Deficits in basic motor and sensory function were tested 24 hours after reperfusion by a blinded examiner using a modified 24-point scoring system [14]. Coronal sections of the brain were stained with 1% 2.3.5-triphenyltetrazolium chloride (TTC, SERVA, Antwerpen, Belgium) for 15 min at 37°C to delineate the ischemic lesion amongst the viable tissue. The hemispheric and infarct volumes of the cortex and striatum were calculated by planimetry of corresponding areas for each slice and thickness (2 mm). Swelling corresponded to the differences between infarcted (left) and non-infarcted (right) hemispheric volumes and infarct volume was corrected for swelling as follows: corrected infarct volume = infarcted volume * (1 - swelling/volume non infarcted hemisphere) [15]. Whereas the corrected cortical and striatal volumes were detailed for each slice, the swelling data were reported as the sum of the segmental volumes. Samples from the cortical penumbra of slice number 3 were used for semi-quantitative real-time polymerase chain reaction analysis (TaqMan, Applied Biosystems, Carlsbad, California, USA) as described previously [13]. Gene expression levels of *interleukin-1ß* (*IL-1β)*, *interleukin-6* (*IL-6), tumor necrosis factor alpha (TNF-α*) and *intercellular adhesion molecule 1* (*ICAM-1*) were calculated as a relative quantity (RQ) according to the ΔΔCt method, which reflects the differences in threshold for each target gene relative to the housekeeping gene *hypoxanthin-guanin-phosphoribosyltransferase (HPRT)* and six sham-operated animals. A previous study demonstrated, that TTC staining at 37°C does not constrict quantitative gene analysis [16]. The concentration of the brain-specific acidic protein S-100ß was determined in serum probes by a commercially available ELISA kit (YK151, Sceti, Tokyo, Japan).

### Statistical analysis

Animals whose infarcted volume lacked cortical involvement were excluded from the analysis, as this phenomenon may be accounted for by residual collateral flow through the circle of Willis. CBF that did not exceed 50% of baseline values during reperfusion was classified as “failed reperfusion” and was excluded from the final analysis [13]. The data were displayed as the mean and standard deviation or as box-and-whisker plots with the 5th and 95th percentile. Differences between the groups were analyzed using an unpaired *t*-test and variance analysis for repeated measurements with the Bonferroni’s post-hoc test. Welch’s correction was performed if variances were significantly different between the groups. Effects on survival were evaluated using a chi-squared test. A one sample *t*-test was used to describe the changes in the RQ values relative to the sham condition (RQ = 1). Statistical significance was set as a p-value of ≤ 0.05 for all statistical analyses (Prism 6, GraphPad Software, La Jolla, California, USA).

## Results

**Figure 1:**
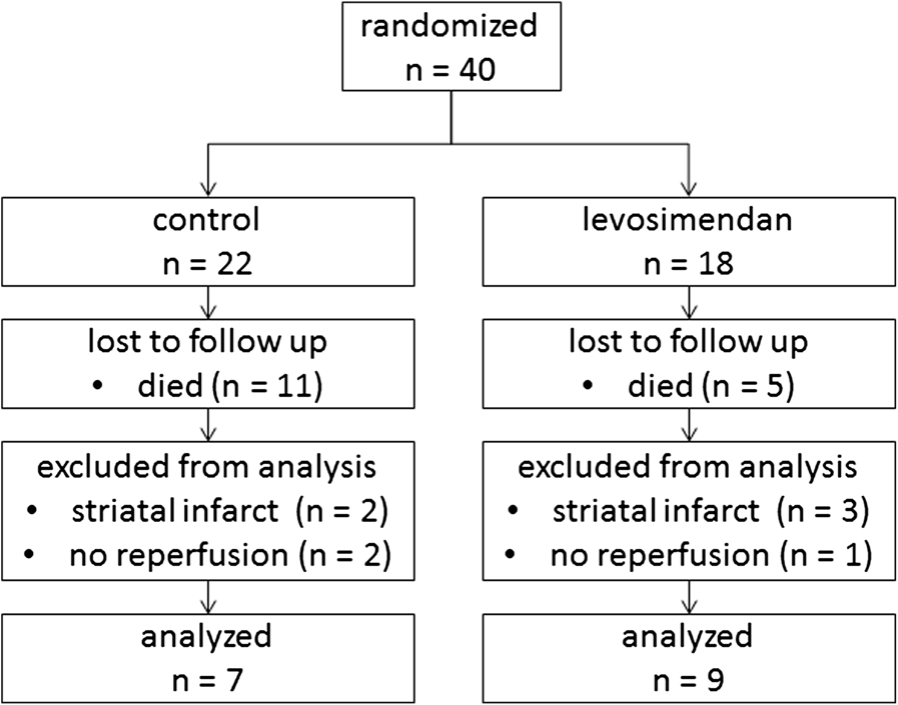
Flow of animals related to stage of experiment.

Twenty-two and 18 animals were assigned to the control group and the levosimendan group, respectively. Eleven controls and 13 levosimendan-treated animals survived 24 hours after reperfusion. Subarachnoid hemorrhage (SAH) led to the death of four animals in the control group. The cause of death for the remaining seven animals could not be determined, but may have been related to a failure of reperfusion. Of the remaining animals, seven in the control group and nine in the levosimendan group demonstrated a cortical infarct after reperfusion. The eight animals with failed reperfusion or lack of involvement of the cortex in the infarcted area were excluded from further analysis. There were no significant differences between the control group and the levosimendan group with respect to survival, infarct pattern or the rate of reperfusion (Figure 1).

### Hemodynamics

**Figure 2:**
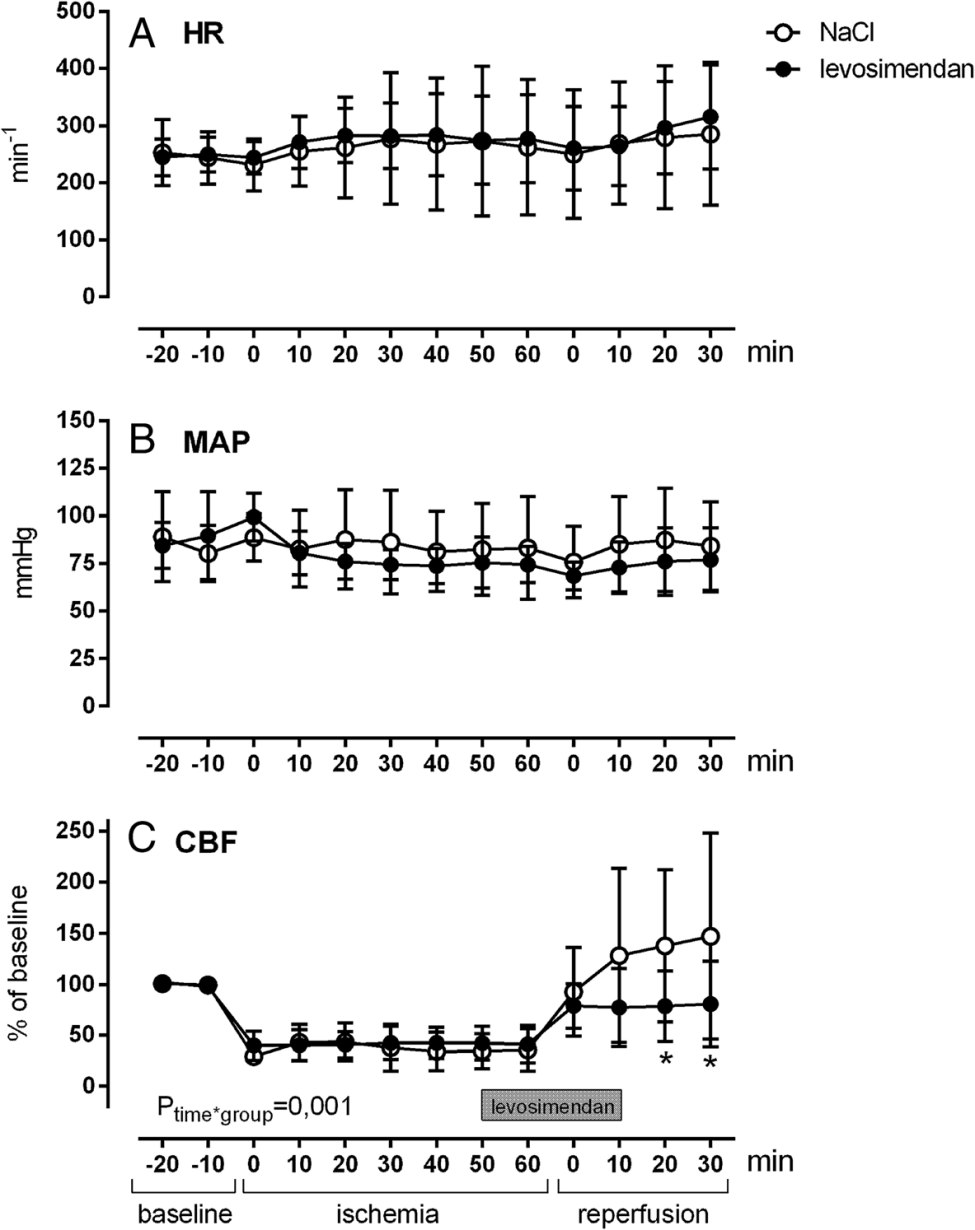
**Effects of transient cerebral ischemia and reperfusion on hemodynamics.** Changes of heart rate (**A,** HR) mean arterial pressure (**B**, MAP) and cerebral blood flow (**C,** CBF) are displayed. Levosimendan infusion over 20 min was started at 5 min before reperfusion (p values from variance analysis for repeated measurements and post hoc test: * p < 0.05 vs. control).

**Figure 3:**
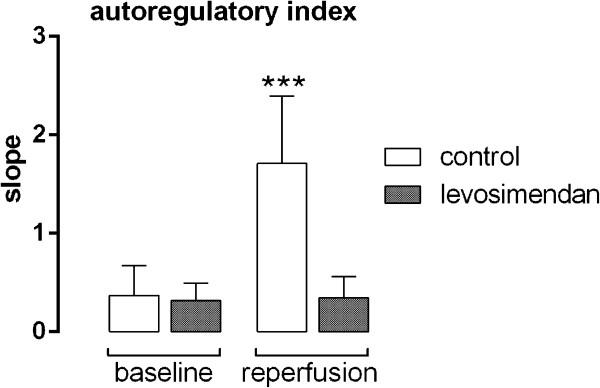
**Effects of levosimendan treatment on the autoregulatory index.** Values after transient MCAO (reperfusion) were compared to values before ischemia (baseline) (*** p < 0.001 vs. levosimendan)**.**

Although HR and MAP did change significantly over time (p = 0.001), no differences between the groups could be observed (Figure 2A/B). There was a significant interaction between time and group for CBF (p = 0.001). Post-hoc analysis showed significantly lower CBF values in the levosimendan group at 20 and 30 min after reperfusion (Figure 2C). ARI demonstrated no differences between the groups (0.37 ± 0.31 vs. 0.32 ± 0.18) before ischemia. During reperfusion, ARI remained unchanged after levosimendan treatment (0.35 ± 0.22; Figure 3) but it increased in the control group (1.71 ± 0.68; p = 0.001), an indication of abrogated autoregulation.

### Neurologic injury

**Figure 4:**
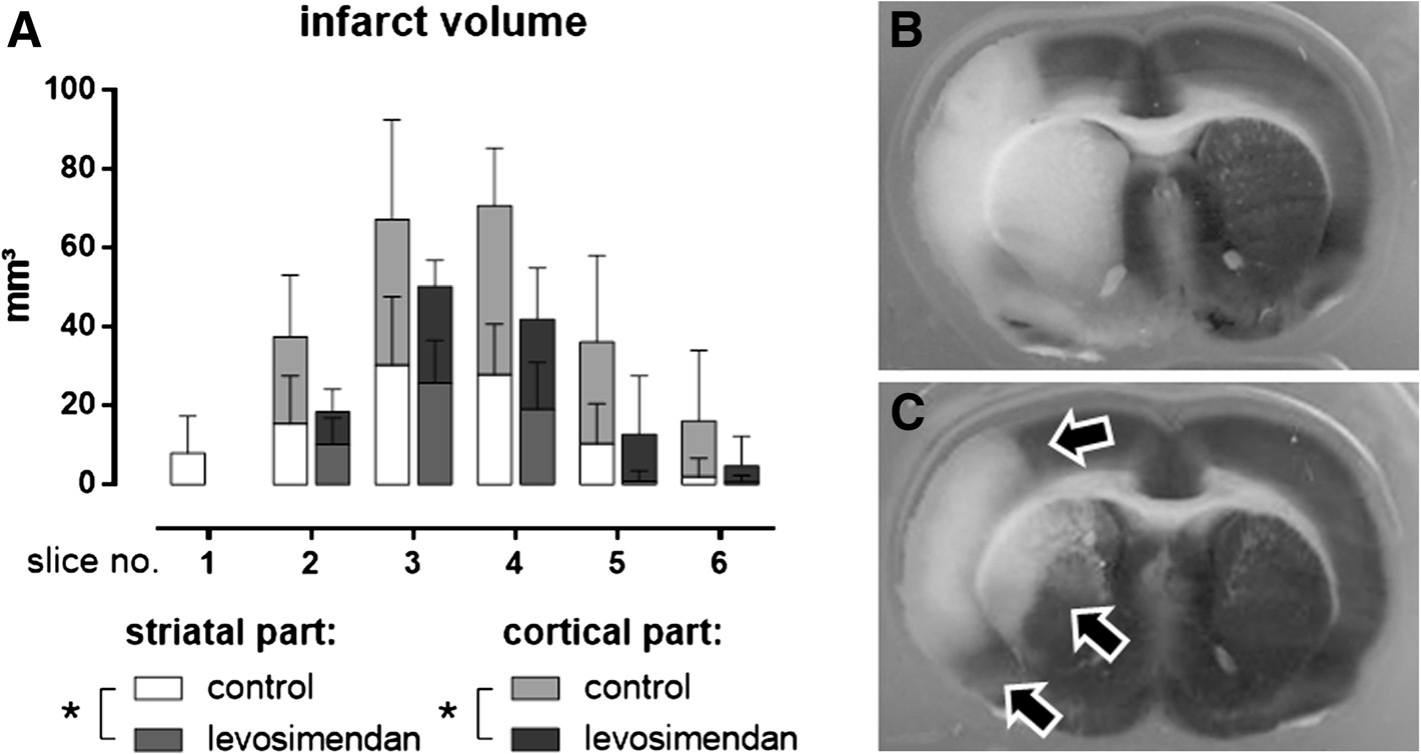
**Effect of levosimendan on cerebral infarct size.** Differences between infarct volume of the striatal and cortical parts of each slice were illustrated **(A)**. A representative slice (slice number 3) after staining with triphenyltetrazolium chloride from a control animal **(B)** and a levosimendan-treated animal **(C)**. The arrows indicate sites of infarct size reduction (* p < 0.05 from analysis of variance for repeated measurements).

**Figure 5:**
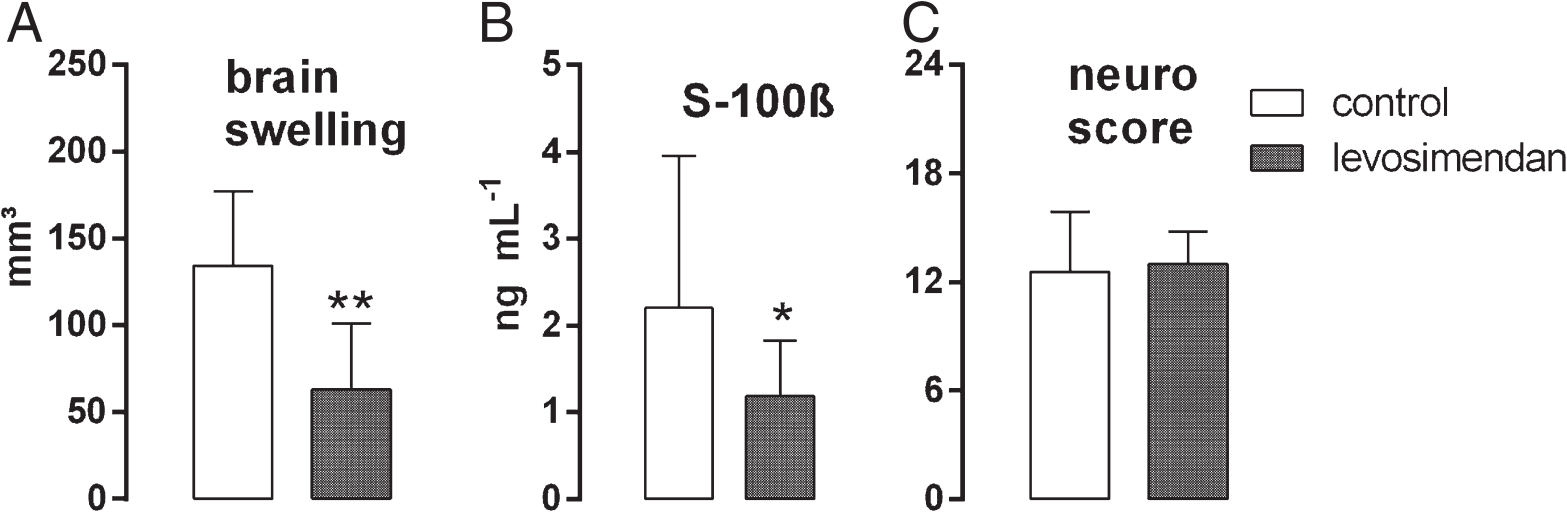
**Additional effects of levosimendan treatment on neurological injury during MCAO.** Differences between groups for brain swelling **(A)**, serum levels of s-100ß **(B)** and neurological testing **(C)** after 24 hours were displayed (* p <0.05 vs. control; ** p < 0.01 vs. control).

The total infarct volume was 239 ± 83 mm^3^ in the control group and 128 ± 42 mm^3^ in the levosimendan-treated group (p = 0.01). This difference was discernible in the cortex (142 ± 79 vs. 72 ± 36 mm^3^; p = 0.03) and the striatum (97 ± 38 vs. 56 ± 21 mm^3^; p = 0.02) in all slices (Figure 4A). Infarct size reduction was noted in the cortex medially and temporally and in the striatum from the center (Figure 4B/C). Total brain swelling was less pronounced after levosimendan treatment compared to the control group (63 ± 38 vs. 134 ± 43 mm^3^; p = 0.004; Figure 5A). Moreover, significantly lower serum levels of s-100ß were measured 24 hours after reperfusion in the levosimendan group versus the control group (1.19 ± 0.64 vs. 2.20 ± 1.75 ng mL^-1^; p = 0.02; Figure 5B). The low neurological test scores attained by both groups are indicative of a relevant functional deficit with no statistically significant differences between the groups (p = 0.12; Figure 5C).

**Figure 6:**
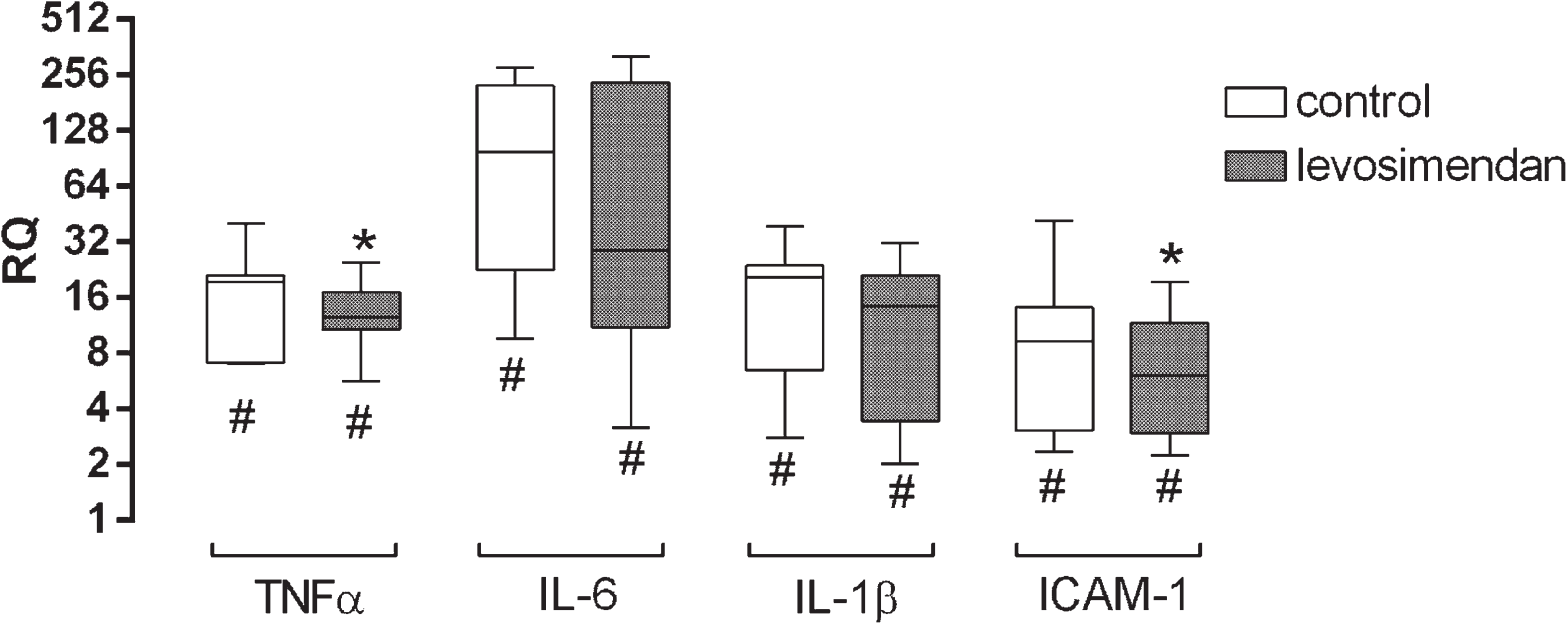
Relative expression of inflammatory markers in the cortical penumbra at 24 hours after MCAO (* p < 0.05 vs. control; ^**# **^**p < 0.05 vs. RQ = 1).**

Expression of inflammatory markers in the cortical penumbra was significantly upregulated (8–18 times) in both groups at 24 hours after MCAO (Figure 6). While RQ values of *TNFα* (p = 0.05) and *ICAM-1* (p = 0.04) remained significantly lower after levosimendan treatment, there were no differences observed in *IL-6* (p = 0.65) and *IL-1ß* (p = 0.53) expression between the groups.

## Discussion

This study is the first to demonstrate a neuroprotective effect of levosimendan in the rat brain using a model of transient MCAO. While structural injury was shown to be reduced, an improvement of neurological outcome could not be demonstrated. Postconditioning with levosimendan reduced infarct size, brain swelling, release of s-100ß, impairment of cerebral autoregulation and the inflammatory response 24 hours after reperfusion.

Transient cerebral ischemia led to an extension of the infarct core despite reperfusion therapy. Although reperfusion restored the energy supply to the neuronal cells of the penumbra, this effect is limited due to the occurrence of secondary energy failure, impairments of the blood brain barrier (BBB), abrogated autoregulation of perfusion and inflammation (reperfusion injury) [3]. Thus, neuroprotective agents may target the neurons or the vasculature of the brain. Activation of mK_ATP_ channels before ischemia (preconditioning) should preserve energy status. In fact, pretreatment with levosimendan has been shown to reduce neuronal injury in traumatic brain injury in vitro [9] and in the rabbit spinal cord in vivo [8]. Postconditioning with levosimendan also improved function and reduced neurological injury in the spinal cord of the rabbit [7]. A similar effect could be achieved with diazoxide in the rat brain after transient ischemia. The reduction of the infarct size was associated with a partial inhibition of mitochondrial permeability transition pore opening, a key mediator of reperfusion injury, without any effect on oxidative phosphorylation [17]. Accordingly, levosimendan failed to improve restoration of metabolism after transient global ischemia in the rat [10]. Thus, improvement of secondary energy failure would not account for the postconditioning effects of levosimendan.

As cerebral injury affects both the integrity of the BBB and autoregulation and because the extent of flow during reperfusion is correlated with neurological injury [18], the current findings that CBF was limited during reperfusion by intact autoregulation may partially explain the neuroprotective properties of levosimendan. A similar effect has previously been demonstrated for nimodipin [19] and magnesium sulfate [20]. K_ATP_ channels have also been shown to play a major role in the autoregulation of CBF [21] and ischemic reperfusion injury has been reported to reduce N-methyl-D-aspartate mediated vasodilatation, which could be prevented by diazoxide [22]. Thus, levosimendan should be able to prevent regional no-reflow and hyperperfusion. Moreover, studies have demonstrated an improvement in cerebral perfusion in critically ill infants with low cardiac output syndrome [23] as well as the prevention of vasospasm in an animal model of subarachnoid hemorrhage [24]. However, improved collateral perfusion by levosimendan could not be demonstrated during ischemia in the current investigation, which may indicate that effects on autoregulation during reperfusion play an important role in this model. However, it remains unclear whether preservation of the external carotid artery may influence these results [25]. Although long-term effects of levosimendan on global hemodynamics, which may influence CBF, cannot be excluded, such effects are rather unlikely after such a short infusion time and have only been described for dosages higher than 36 μg/kg [26].

Additionally, the activation of K_ATP_ channels should reduce the permeability of the BBB and thus reduce cerebral edema and inflammation [3,27]. Indeed, the results show that levosimendan reduced brain swelling and the expression of *TNFα* and *ICAM-1*. This effect could not be demonstrated after transient global cerebral ischemia [10]. As brain injury triggers the inflammatory response and aggravates the injury [28], the decreased expression of *TNFα* and *ICAM-1* indicate a diminished progression of injury. Cytokines such as *TNFα* will stimulate expression of *ICAM-1*, leading to leukocyte adhesion and extravasation [29].

In summary, it is difficult to determine which mechanism mediates the neuroprotective action of levosimendan. Although only low tissue concentrations will have reached the brain with a single bolus of 24 μg kg^-1^ levosimendan [10], it is reasonable to assume that it has a direct effect on neuronal cells, as expansion of the infarct core from the lateral cortex and lateral striatum reflects the early stages (4–8 hours) after untreated MCAO [30]. It is nearly impossible to distinguish whether the vascular effects of levosimendan are a consequence of reduced brain injury or a primary effect. The inconsistent effects of levosimendan reported in different studies may be explained by the differences in the pathomechanisms and in the extent of neuronal injury [10]. The aim of the current protocol for levosimendan was to reach the highest possible serum concentrations during early reperfusion with limited hemodynamic side effects. In contrast, a delayed and extended application may result in higher effect-site concentrations and pronounced differences.

In the current investigation, postconditioning with levosimendan led to a 46% reduction in infarct volume, which concurs with the findings of other studies (hypothermia = 40%, ischemic postconditioning = 40%, diazoxide = 54%) [3,17]. This effect may be too small to allow for the detection of functional effects. Nevertheless, several studies have demonstrated significant effects of neuroprotective interventions on functional recovery [31-33], and a review article reported that at least 80% of the integrity of the ipsilateral hemisphere should be preserved to provide partial or complete neurologic recovery [34]. This range could have been closely approximated by the inhalation of the noble gas xenon [35]. However, the Stroke Therapy Industry Roundtable (STAIR) recommends that a longer observational period of at least three days along with repetitive sophisticated testing is more important in describing neurological deficits [36]. Even pronounced recovery within this first week in untreated animals should be noted [37].

Several limitations related to the study design warrant mention. No tissue samples were collected to analyze protein expressions and no further techniques were used to quantify cell death in the cortex and the hippocampus. The exclusion of 24 animals by the predefined criteria resulted in a small number of animals per group. The high mortality rate deserves further discussion. A current review calculated the average mortality in rat stroke experiments as 15%, with a range between 0% and 60% [38]. SAH and failed reperfusion, which could lead to larger infarcts, may account for the high mortality rate observed in the current study and may be related to the shape of the filament.

Further studies are needed to determine the sites of action and the feasibility of levosimendan as a neuroprotective agent. Possible targets may include the reduction of cerebral injury after resuscitation, ischemic stroke or cerebral vasospasm [24]. The cardioprotective properties may also be of interest as brain injury may induce heart failure [39].

## Conclusions

In conclusion, the application of levosimendan during ischemia and early reperfusion limited cerebral reperfusion injury. Although infarct size, brain swelling, inflammation and impairment of autoregulation were reduced, no effect on mortality or neurological function could be observed.

## Competing interests

The authors declare that they have no competing interests.

## Authors’ contributions

MH, NZ and AR conceived of the study, participated in the study’s design and coordination, performed the statistical analysis and drafted the manuscript. AR, CB and MH conducted the experimental laboratory work. CB and RR assisted in drafting the manuscript. All authors read and approved the final manuscript.

## Pre-publication history

The pre-publication history for this paper can be accessed here:

http://www.biomedcentral.com/1471-2377/13/106/prepub
